# Complete Genome Sequence of a Porcine Endogenous Retrovirus Isolated from a Bama Minipig in Guangxi, Southern China

**DOI:** 10.1128/genomeA.00620-15

**Published:** 2015-06-11

**Authors:** Hai-bo Tang, Kang Ouyang, Ling Ma, Anbin Bai, Shaomin Qin, Fenglian Chen, Jun Lin, Yinyin Cao, Jianmin Wu

**Affiliations:** Guangxi Veterinary Research Institute, Nanning, China

## Abstract

A porcine endogenous retrovirus (PERV) strain, PERV-A-BM, was isolated from a Bama minipig in Guangxi, China. This is the first entire genome sequence of PERV isolated from Guangxi Bama minipigs. The isolate is closely related to isolates from Wuzhishan miniature pigs and distantly related to isolates from large white pigs.

## GENOME ANNOUNCEMENT

Porcine endogenous retroviruses (PERVs) are a major concern when porcine tissues and organs are used for xenotransplantation (1), because there are reports that they can infect human cells *in vitro*, and their pathogenic potential is unknown in cross-species transmission. Guangxi Bama minipigs are potential organ donors for xenotransplantation in China. So far, however, information on the molecular characteristics of PERV from Guangxi Bama minipigs has not been sufficient. We therefore investigated characteristics in the Guangxi Bama minipigs in order to further characterize the risks associated with PERV transmission.

A PERV strain was isolated in 2010 from peripheral blood mononuclear cells of a Bama minipig in Guangxi, China, which was designated PERV-A-BM. To clarify the characteristics of the virus, total RNA was extracted from the peripheral blood mononuclear cells by using the TRIzol LS reagent (Invitrogen, Carlsbad, CA). Three pairs of primers and one pair of nested-PCR primers (rapid amplification of cDNA 3′ ends [3′ RACE]) for amplifying the whole PERV genomes were designed based on the genomic sequence of strains (GenBank accession numbers AF435966 and HQ540592) (2, 3). PCR products were purified using the Aqua-SPIN gel extraction minikit (Tiangen, Inc.) and cloned into a pMD18-T vector (TaKaRa, Inc.). The cloned DNA fragments were then sequenced by Sangon Biotech (Shanghai Co., Ltd.) The genomic sequence was assembled via the SeqMan software (DNAStar, Inc.).

The complete genome of strain PERV-A-BM is 8,774 nucleotides in length, with a G+C content of 50.17%. The lengths of the coding sequences are as follows: 1,575 nucleotides (nt) for gag, 3,438 nt for pol, and 1,965 nt for env. The 5′ untranslated region (UTR) was nucleotides 1 to 1070, consisting of a leader sequence and long terminal repeats (LTRs), and the LTRs consist of U3, R, and U5 regions, which have been identiﬁed in previous research (4). The length of the 5′ LTR was 553 nt, and the 3′ UTR was 642 nt. Three replication-competent classes of PERVs (PERV-A, -B, and -C) have been identiﬁed in the genomic DNA of pigs and porcine cell lines (5, 6). A sequence comparison indicates that the isolate belongs to the subgroup class A, which includes strains isolated from South Korean native pigs (3). The isolate is closely related to the strain isolated from Wuzhishan miniature pigs in Hainan Province, China (homology, 97.1%) (7) and distantly related to strains isolated from large white pigs (GenBank accession numbers EU789636 and AJ279057) (homology, 90.0 to 90.7%) (2). Although the virus was isolated from a Guangxi Bama minipig, the analysis and identification of its complete genome will be of help to further reveal the characteristics of the retrovirus genus of PERV from different *Sus scrofa* breeds.

### Nucleotide sequence accession number. 

The complete genome sequence of PERV-A-BM is available in GenBank under the accession no. HM159246.
